# The endothelin B (ET_B_) receptor agonist IRL 1620 is highly vasoconstrictive in two syngeneic rat tumour lines: potential for selective tumour blood flow modification

**DOI:** 10.1038/sj.bjc.6602672

**Published:** 2005-06-21

**Authors:** M Cemazar, I Wilson, V E Prise, K M Bell, S A Hill, G M Tozer

**Affiliations:** 1 Gray Cancer Institute, Mount Vernon Hospital, PO Box 100, Northwood, Middlesex HA6 2JR, UK

**Keywords:** rat carcinosarcoma, endothelin-1, ET_B_ agonist IRL 1620, ET_A_ and ET_B_ receptors distribution, blood flow, vascular resistance

## Abstract

The vascular effects of the endothelin B (ET_B_) receptor agonist IRL 1620 were investigated in the rat P22 carcinosarcoma and a range of normal tissues in BDIX rats. Tissue blood flow rate was calculated from measurements of tissue uptake of radiolabelled iodoantipyrine. A comparison of vascular effects in the P22 tumour and the HSN sarcoma growing in CBH/CBi rats was made using laser Doppler flowmetry, showing similar effects of IRL 1620, with red cell flux rapidly decreasing by 50–60% and then returning to control levels within approximately 30 min. This corresponded to similar levels but different spatial organisation of ET_B_ binding sites in the two tumours, as measured by autoradiography. The decrease in tumour blood flow and an increase in vascular resistance suggest that the vascular component of ET_B_ receptors in the P22 tumour is localised on contractile elements rather than on endothelial cells. ET_A_ receptors were also identified. Vasoconstriction occurred uniformly throughout the P22 tumour mass, consistent with a measured homogeneous distribution of ET_B_ receptors. IRL 1620 caused vasoconstriction in normal skeletal muscle, kidney and small intestine of the BDIX rat as well as in tumour, but did not affect blood flow in other tissues. These effects could be useful for limiting toxicity of certain chemotherapeutic agents. Fully functional ET_B_ receptors are clearly expressed on tumour vasculature and IRL 1620 shows promise for short-term modification of tumour blood flow. Expression levels of ET_B_ receptors on the tumour vasculature could be useful for predicting which tumours are likely to respond to IRL 1620.

Tumour vasculature is morphologically and functionally abnormal (Carmeliet and Jain, 2000). Therefore, the vascular reactivity of tumours differs from that of normal tissue, providing the potential for selective modification of tumour blood flow for therapeutic benefit (Brown and Giaccia, 1998). Increase in blood flow can elevate the concentration of antitumour agents reaching tumour cells and improve oxygenation, with consequent benefit for both chemotherapy and radiotherapy (Chaplin *et al*, 1998). However, most success in this field has come from strategies designed to damage the tumour vasculature selectively, leading to sustained blood flow shut-down and consequent tumour cell death (Thorpe *et al*, 2003). In addition, vascular dilatation is one of the earliest steps in angiogenesis (Hudlicka, 1998), so that vasoconstrictors have potential as antiangiogenic agents. A strategically timed reduction in blood flow can effectively trap anticancer agents in tumour tissue (Pedley *et al*, 2001) and the reduced oxygenation that accompanies blood flow reduction has potential for activating the so-called ‘bioreductive’ drugs (Brown, 2002). In order to control tumour blood flow for therapeutic purposes effectively, it is essential to understand the mechanisms controlling vascular tone in tumours.

Endothelin-1 (ET-1), together with ET-2, ET-3 and the ET receptors (collectively called the endothelin axis), exerts many important physiological roles in normal tissues, as well as in cancer (Nelson *et al*, 2003). Endothelin-1 was first described as a potent endogenous vasoconstrictor peptide, but is now also known to play important roles in tissue differentiation, repair, development, cell proliferation, hormone production, angiogenesis, matrix remodelling and apoptosis. It is primarily produced by endothelial, vascular smooth muscle and epithelial cells and has also been detected in many different tumours (Bagnato *et al*, 1999; Kurbel *et al*, 1999; Nelson *et al*, 2003).

The vasoactive action of ET-1 is mediated primarily via endothelin A (ET_A_) and endothelin B (ET_B_) receptors. ET_A_ receptors are mainly found on vascular smooth muscle cells and pericytes and these account for the profound vasoconstriction of ET-1. ET_B_ receptors are found on contractile elements and endothelial cells and their activation can cause both vasoconstriction (activation of receptors on vascular smooth muscle cells/pericytes) and vasodilatation (activation of receptors on endothelial cells) (Davenport, 2002).

Tumour vasculature is often rather insensitive to vasoactive agents, presumably due either to a relative lack of vascular smooth muscle cells supporting the tumour vasculature, a lack of functional receptors, low binding affinities or lack of downstream effector mechanisms. However, very few studies have investigated these factors directly. The endothelin receptor family represents a group of receptors, which has a profound role in the control of normal tissue blood flow under both physiological and pathological conditions (Haynes and Webb, 1994; Russell *et al*, 1998; Stjernquist, 1998) and which may be possible to exploit for selective modification of tumour blood flow (Bell *et al*, 1996, 1997, Rai and Gulati, 2003; Sonveaux *et al*, 2004). The development of highly specific agonists and antagonists for ET-1 receptors has provided tools for studying the role of ET-1 in tumour vasculature. In our previous study on the HSN rat fibrosarcoma, radiotracer techniques were used to determine blood flow in tumour and selected normal tissues following systemic treatment with the selective ET_B_ receptor agonist IRL 1620. Surprisingly, the HSN tumour vasculature constricted relatively selectively in response to this agonist compared with the majority of normal tissues, with the exception of the small intestine (Bell *et al*, 1999). This unusual feature of IRL 1620 warranted its study in other tumour types. In an additional preliminary study using autoradiography in the HSN tumour, we showed that there were small regions of intense specific ET_B_ binding sites scattered throughout the tumour mass, which were suggestive of a vascular localisation of ET_B_ receptors in this tumour type (Bell *et al*, 1998).

The main aim of the current study was to continue evaluation of IRL 1620 as a selective tumour blood flow modifier by extending studies to a second tumour type (P22 rat carcinosarcoma) and investigating a full time course of vascular response in tumours *vs* normal tissues. Furthermore, the relationship between tumour vascular response and degree of specific binding sites for IRL 1620 in tumour tissue was investigated. Laser Doppler flowmetry was used to compare directly the time course of vascular response in the P22 and HSN tumours.

## MATERIALS AND METHODS

### Animals and tumours

Early generations of the P22 transplanted rat carcinosarcoma were grown subcutaneously in the left flank of 10- to 12-week-old male BDIX rats as described previously (Tozer and Shaffi, 1993). Tumours were used for experiments when they reached 1–2 g (all three orthogonal diameters 10–15 mm, including the skin thickness). Laser Doppler flowmetry studies were also carried out on early generations of the rat HSN fibrosarcoma grown in the flank of 8- to 10-week-old female CBH/CBi rats (Bell *et al*, 1999). Tumours were used for experiments when they reached 1–2 g in weight. All experiments were carried out in accordance with the UK Animal (Scientific Procedures) Act 1986.

### Drugs

IRL 1620 ([suc-(Glu^9^, Ala^11,15^)-ET-1(8–21)]), (Alexis, Nottingham, UK) was dissolved in physiological saline and administered to the rats by slow bolus intravenous (i.v.) injection, at a dose of 3 nmol kg^−1^ in an injection volume of 4 ml kg^−1^, which was an effective dose in the HSN tumour model (Bell *et al*, 1999).

### Laser Doppler flowmetry

Red blood cells (RBC) flux was monitored using the Oxford Array multiple channel laser Doppler system (Oxford Optronics, Oxford, UK). Rats were anaesthetised by intraperitoneal (i.p.) injection of fluanisone (10 mg kg^−1^), fentanyl citrate (0.315 mg kg^−1^) (‘Hypnorm’, Janssen Animal Health, UK) and midazolam (2 mg kg^−1^) (‘Hypnovel’, Roche Products Ltd, UK) and kept warm using a thermostatically controlled heating pad. Five microprobes were randomly inserted into each tumour through skin incisions, to a depth of at least 1 mm. After a 20 min stabilisation period, animals were treated with IRL 1620 and RBC flux was monitored continuously for 70 min. Measurements were continued for at least a further 5 min following a lethal injection of anaesthetic to allow the biological zero RBC flux to be measured. From the 20 readings per second recorded, 3 min averages were calculated for each probe. After the subtraction of biological zero, the data were converted to the relative RBC flux, expressed as the percentage of the RBC flux at time 0 (time of injection of IRL 1620). Mean values for each animal at each time point were calculated from the individual probe values and these were used to calculate the overall mean value for relative RBC flux, at each time point, for the whole treatment group.

### Blood flow

The experimental procedure for the determination of blood flow using ^14^C-labelled iodoantipyrine (^14^C-IAP) (Amersham Life Sciences, UK) and ^125^I-labelled iodoantipyrine (^125^I-IAP) (Institute of Cancer Research, Sutton, UK) has been described previously (Tozer and Shaffi, 1993). In brief, rats were anaesthetised and kept warm, as described above. One tail artery and two tail veins were catheterised with polyethylene catheters containing heparinised 0.9% saline. Mean arterial blood pressure (MABP) was recorded throughout by means of a physiological pressure transducer (Ohmeda, UK) connected to the arterial line.

At different time points (5, 15, 20, 30 min) before the determination of blood flow, a 3.0 nmol kg^−1^ bolus i.v. injection of IRL 1620 or saline (drug vehicle in control rats) was administered to the rat. The rats were heparinised 5 min prior to blood flow measurement via a venous catheter (0.1 ml of 1000 U ml^−1^ heparin (CP Pharmaceuticals Ltd, UK)). Free flowing arterial blood was collected into preweighed vials at 1 s intervals, during a 30 s i.v. infusion of 0.56 MBq (15 *μ*Ci) ^14^C-IAP or 0.37 MBq (10 *μ*Ci) ^125^I-IAP prepared in 0.9% saline. Infusion was achieved via a constant flow infusion pump set at a rate of 1.6 ml min^−1^, which compensated for the blood loss from the arterial circulation and maintained blood pressure constant (unpublished data). At 30 s, the rat was rapidly killed by i.v. injection of Euthatal (0.3 ml) (Rhône Mérieux Ltd, UK) and the tumour and selected normal tissues were rapidly excised. When using ^125^I-IAP, blood and tissues were weighed and the ^125^I levels determined using a Wallac (Bromma, Sweden) Autogamma well counter. In the case of ^14^C-IAP blood flow measurements, no normal tissues were excised. Blood samples were weighed and 10 *μ*l aliquots were placed in liquid scintillation vials to which 1 ml Soluene (Canberra-Packard, UK) was added. Vials were left overnight, and then 10 ml scintillation fluid (Hionic-Fluor, Canberra-Packard, UK) was added. Radioactivity was determined by liquid scintillation counting (Beckman LSG 500 multi-purpose scintillation counter, Fullerton, CA, USA) with suitable quench correction. Immediately after excision, the tumours were mounted in Tissue-Tek embedding compound (Raymond Lamb, UK), frozen in isopentane at −40°C and stored at −70°C for subsequent processing. Cryostat sections (20 *μ*m thick) were cut from each tumour, mounted onto poly-L-lysine-coated slides (Sigma, Poole, UK) and rapidly dried on a hot plate. Slides were overlaid with autoradiographic film (Hyperfilm *β*max, Amersham, UK) for up to 2 weeks, alongside ^14^C-labelled methylmethacrylate microscales (Amersham, UK) of known isotope concentration for quantification of autoradiograms. Following development of autoradiograms, sections were fixed in methanol and stained with haematoxylin and eosin (H&E).

### Analysis of blood flow results

Blood flow to the tumour and normal tissues was calculated in ml g^−1^ min^−1^ from the tissue counts measured via either autoradiography or gamma counting, the equilibrium partition coefficient of IAP in the different tissues and the arterial input function derived from the arterial blood counts. The mathematical model used was adapted from Kety (1960) and is described elsewhere in detail (Tozer *et al*, 1994). Perfusion pressure for all tissues was assumed to change in direct proportion to MABP, and changes in tissue vascular resistance (TVR) following treatment, for the tumour and normal tissues, were calculated from the changes in MABP divided by the changes in blood flow rate.

A single blood flow value was obtained for each tissue, where counts were obtained by gamma counting. Autoradiographic images were captured by camera and transformed pixel by pixel into blood flow rate images using the same principles as described above. Analysis was performed using in-house software developed using the Visilog Image Processing package (Noesis, Orsay, France). Blood flow autoradiogram images were overlaid onto histological images from H&E-stained sections to exclude necrotic regions from the calculations of blood flow. Tumour peripheral regions were defined as the rim of the tumour equal to 10% of the section diameter, the remaining portion of the section being defined as the central region.

### Spatial distribution of ET-1 receptors

Distribution of ET_A_ and ET_B_ receptors in untreated P22 tumours was assessed on sections of the same tumours as used for the blood flow determination. Preliminary experiments were carried out to determine suitable incubation concentrations of compounds and suitable incubation and washing times to maximise specific binding (data not shown). Slides containing serial pairs of tumour sections were incubated in wash buffer (50 mM Tris/HCl buffer, pH 7.4, containing 100 mM NaCl, 5 mM MgCl_2_, 40 mg l^−1^ bacitracin, 1% BSA) (15 min, room temperature) to remove endogenous peptides, drug and ^14^C-IAP. Sections were dried at room temperature in air, then incubated for 90 min with ^125^I-ET-1 (0.13 nM) plus phosphoramidon (to inhibit action of endothelin/converting enzyme; 1 *μ*M) (a) alone (to give total binding ET_A_+ET_B_), or with the addition of either (b) BQ-788 (ET_B_ receptor antagonist; 100 nM; to give total ET_A_ binding), or (c) BQ-610 (ET_A_ receptor antagonist, 100nM; to give total ET_B_ binding). The further inclusion of cold ET-1 (1 *μ*M) to each group allowed the determination of nonspecific binding in each case. After incubation, sections were rinsed three times in wash buffer, dried in air and exposed to Hyperfilm-^3^H (Amersham) for 24 h alongside ^125^I-microscales. Hyperfilm-^3^H has no scratch-resistant coating and is therefore suitable for detection of Auger electrons, which are emitted in addition to gamma-rays during the decay of ^125^I. Exposure of test sections for varying times showed that 24 h was the optimum time for obtaining grey levels that covered the dynamic range of the film. Autoradiograms were developed and sections fixed in methanol and stained with haematoxylin and eosin.

### Quantitation of receptor binding

Levels of radioactivity in the ^125^I-microscales were converted to total and nonspecific binding (fmol mg^−1^ protein) using the specific activity of the ^125^I-microscales supplied by the manufacturer (Amersham). These modified scales were used to generate standard curves from which optical density of the images of the tumour sections were transformed into total, specific and nonspecific binding images. Specific binding for each pair of sections used for receptor studies was calculated by subtraction of the mean nonspecific binding from the mean total binding using the whole sections as the region of interest. Image analysis was carried out using in-house software developed using the Visilog Image Processing package.

### Statistical analysis

The radiotracer blood flow data were tested for normality of distribution. Differences between groups were tested using a one-way analysis of variance (ANOVA) followed by a Tukey–Kramer post-test; *P*-values less than 0.05 were taken as indicative of significant differences between groups. Data are presented as arithmetic means±s.e. (standard error of the mean). Temporal blood flow changes for the radiotracer data were analysed using linear regression and the computed curves were compared for significance using ‘overall test for coincidence of the two regression lines’ (Glantz, 2002). Laser Doppler data were fitted to a multivariate model (MANOVA) with repeated measures, in order to determine the effect of treatment. Responses were fitted to effects using least squares. Differences in responses due to treatment or time were tested for significance using an approximate F-test (JMP Statistics Version 5 for the Apple Macintosh, SAS Institute Inc., Cary, NC, USA). Differences in response were described as significant, if the probability corresponding to the F-value was < 0.05.

## RESULTS

### RBC flux

**Figure 1:**
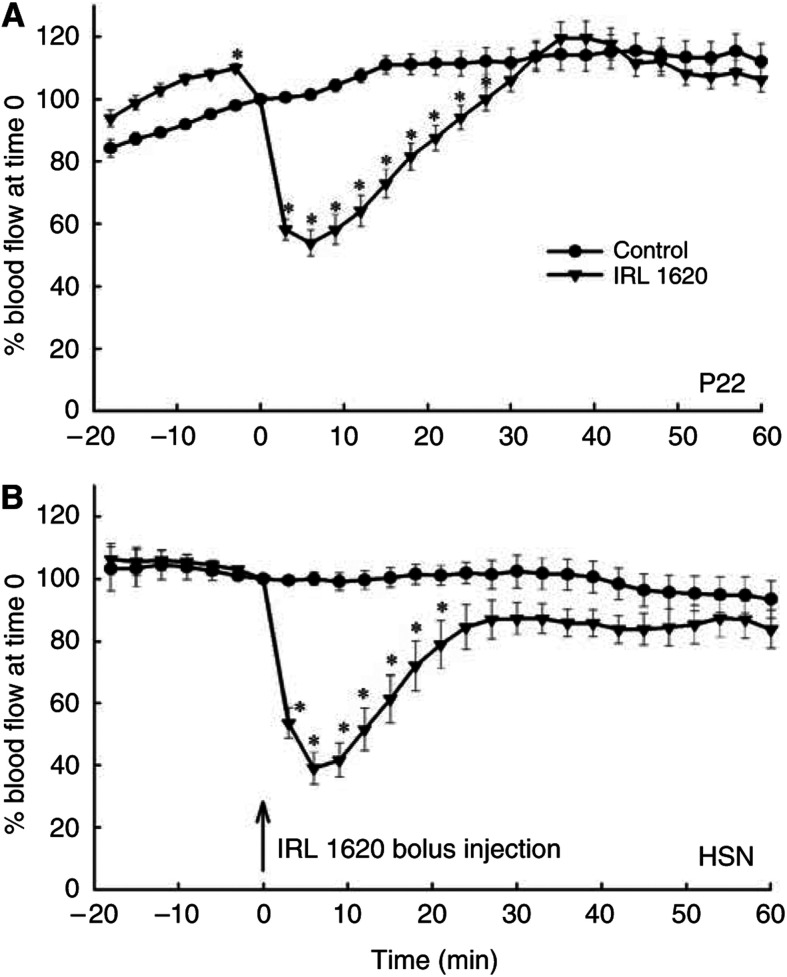
Time course of relative RBC flux changes after i.v. treatment with IRL 1620 (3 nmol kg^−1^) (100% is the value in control tumours at the time of IRL 1620 injection) in P22 (**A**) and HSN (**B**) tumours. Each point represents arithmetic mean±s.e. for six animals. A significant difference from control is represented by (^*^).

Treatment with IRL 1620 at a dose of 3 nmol kg^−1^ decreased RBC flux immediately after injection, as measured by Laser Doppler flowmetry in both tumour models (Figure 1). The response pattern was similar in the two tumours, with the effect most pronounced at 5–10 min after the IRL 1620 injection. RBC flux in P22 tumours was decreased by 50% and in HSN tumours by 60% of pretreatment values and the recovery to pretreatment value was somewhat quicker in HSN tumours (approximately 25 min following IRL 1620 injection for HSN compared to approximately 35 min for P22 tumours).

### Blood pressure and heart rate (HR)

**Figure 2:**
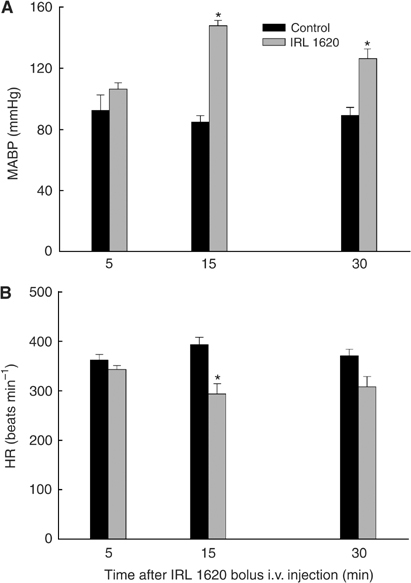
Changes in mean arterial blood pressure (**A**) and HR (**B**) of P22 tumour-bearing control-untreated and IRL-treated rats. Each bar represents arithmetic mean±s.e. for three to six animals. A significant difference from control is represented by (^*^).

Mean arterial blood pressure and HR of anaesthetised BDIX tumour-bearing rats were measured simultaneously with blood flow determinations. Mean arterial blood pressure was significantly increased 15 and 30 min after IRL 1620 administration, with no significant change at 5 min (Figure 2A). The opposite pattern was observed for HR, with a significant effect only occurring at 15 min after treatment (Figure 2B).

### Blood flow and vascular resistance

Absolute blood flow to the P22 tumour and a range of normal tissues in anaesthetised saline-treated BDIX rats is presented in Table 1. Tumour blood flow was similar to that of blood flow to muscle, lower compared to that of brain, kidney, spleen and small intestine, but higher compared to that of skin. Blood flow to skin overlying tumour was significantly higher than that to contralateral skin, which indicates that tumour has a significant vascular influence on surrounding tissue.

**Figure 3:**
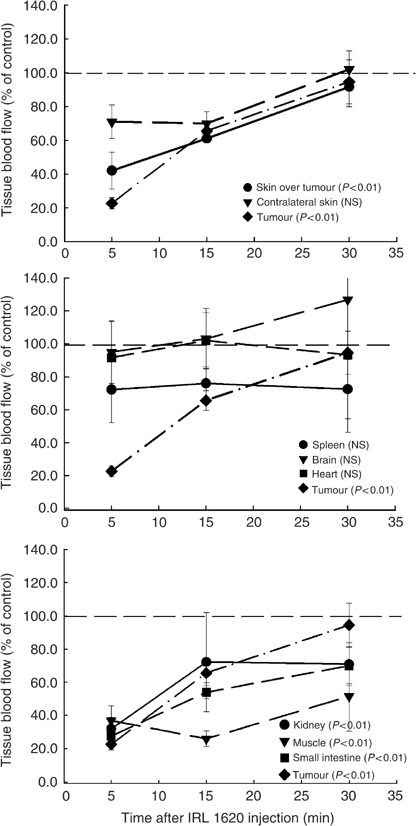
Time course of relative blood flow changes in P22 tumour and normal tissues of BDIX rats following i.v. administration of IRL 1620 at a dose of 3 nmol kg^−1^ plotted as % of the value in time-matched saline-treated rats. Each time point represents arithmetic mean±s.e. for three to six animals. *P*-values represent significance levels for statistical comparisons of curves obtained from drug-treated and saline-treated rats (see Materials and Methods for details).

A similar pattern of blood flow response in the P22 tumour was obtained using uptake of ^125^I-IAP as for RBC flux (comparison of Figures 1 and 3). Tumour blood flow following IRL 1620 administration was reduced to 20% of the control value by 5 min after treatment (Figure 3). Thereafter, tumour blood flow slowly recovered to 65% of control blood flow at 15 min, and to control values at 30 min after treatment. IRL 1620 had no significant effects on blood flow to brain, heart, contralateral skin and spleen. That is, regression lines fitted to experimentally obtained blood flow data from saline-treated control animals and animals treated with IRL 1620 did not differ for these tissues (NS in Figure 3). Blood flow to tissues in saline-treated rats did not change significantly over the time course of the experiments (data not shown). Reduction in flow to skin overlying tumour relative to controls was small, but statistically significant. The most sensitive normal tissues were skeletal muscle, kidney and small intestine. Blood flow to muscle was reduced to ∼40% and remained at that level 30 min postdrug administration. Responses of kidney and small intestine to treatment with IRL 1620 were similar to that of tumour, although recovery was less apparent, with blood flow to these two tissues remaining at ∼70% of control blood flow at 30 min postdrug (Figure 3).

**Figure 4:**
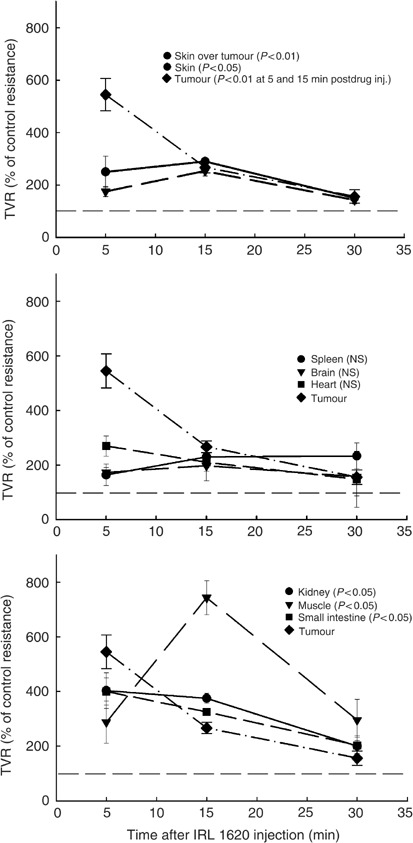
Time course of TVR changes in P22 tumour and normal tissues of BDIX rats following i.v. administration of IRL 1620 at a dose of 3 nmol kg^−1^ plotted as % of the value in time-matched saline-treated rats. Each time point represents arithmetic mean±s.e. for three to six animals. *P*-values represent significance levels for statistical comparisons of curves obtained from drug-treated and saline-treated rats (see Materials and Methods for details).

Changes in vascular resistance were calculated for tumour and all normal tissues (Figure 4). Tumour vascular resistance at 5 min postdrug injection increased by a factor of 5.5 and was greater than that achieved in any normal tissues at that time. Thereafter, tumour vascular resistance decreased and reached control values at 30 min postdrug injection. Treatment of animals with IRL 1620 did not change the vascular resistance in brain, heart and spleen compared to control saline-treated animals. Vascular resistance in kidney and small intestine was increased at all time points, by ∼4-fold at 5 and 15 min and two-fold at 30 min following IRL 1620 administration. At all time points, there was a small, but significant increase in vascular resistance in skin overlying tumour and contralateral skin. Vascular resistance in the skeletal muscle was also increased at all time points, with a maximum increase (7.5-fold) at 15 min following IRL 1620 administration, which was much greater than any other tissue, including the tumour, at this time point. This difference in time course of vascular effect in skeletal muscle implies different binding kinetics in this tissue compared with the other tissues studied.

### Spatial distribution of blood flow

**Figure 5:**
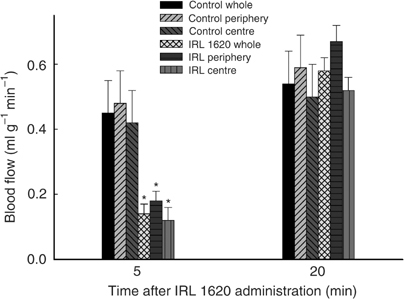
Absolute blood flow across tumour section at 5 and 20 min following i.v. treatment with IRL 1620 at a dose of 3 nmol kg^−1^. Each bar represents arithmetic mean±s.e. for three to six animals. A significant difference from pertinent control (whole, periphery or centre) is represented by (^*^).

**Figure 6:**
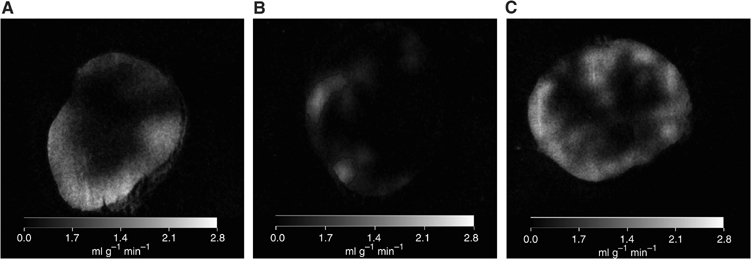
Representative computed images of control P22 tumour blood flow (**A**), at 5 min (**B**) and 20 min (**C**) following i.v. treatment with IRL 1620 at a dose of 3 nmol kg^−1^.

Spatial distribution of tumour blood flow was determined in control tumours and 5 and 20 min following IRL 1620 administration (Figures 5 and 6). Blood flow to tumours from control saline-treated animals tended to be higher at the edge compared to the centre of tumour (Figure 6), but when analysed using our definitions of periphery and centre (Materials and Methods and Figure 5), this difference was not statistically significant. Treatment with IRL 1620 reduced blood flow uniformly throughout the tumour at 5 min post-treatment with a return to control values by 20 min (Figures 5 and 6). Tumour blood flow changes in frozen tumour sections did not entirely follow the changes observed in RBC flux and tumour blood flow measured by the ^125^I-IAP method. Tumour blood flow, measured by autoradiography 5 min after drug administration, was reduced to 25% of control blood flow, which is in accordance with the data obtained by the ^125^I-IAP uptake method. However, this is a larger effect than that measured by Laser Doppler flowmetry, which showed that RBC flux was reduced to 50% of the pretreatment values at this time. This difference may reflect the fact that the IAP technique is primarily measuring plasma flow rather than RBC flux, although this explanation remains speculative in the absence of data from a direct comparison of these techniques. In contrast, 20 min after treatment, the blood flow measured on frozen tumour sections had returned to control values, while RBC flux and blood flow measured with ^125^I-IAP method were still at ∼70% of pretreatment value. It is difficult to provide an explanation for this difference apart from invoking intertumour heterogeneity of response, perhaps related to the different tumour transplantations used in the various experiments.

### Receptor distribution

**Figure 7:**
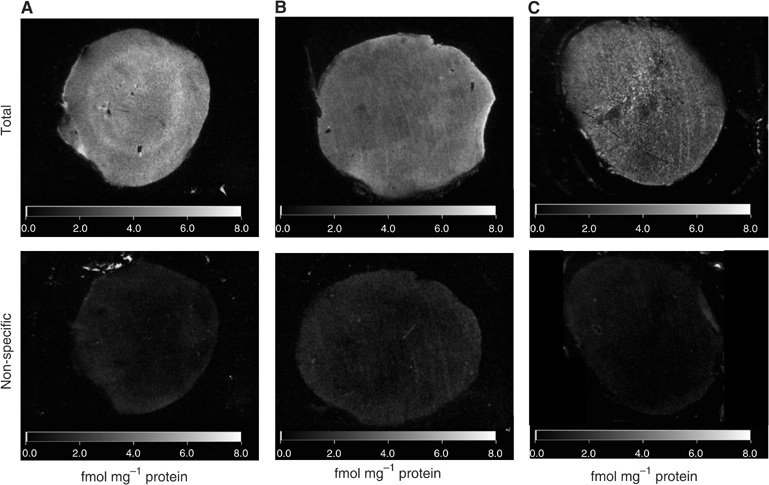
Representative computed images of total and non-specific ET_A+B_ (**A**), ET_A_ (**B**) and ET_B_ (**C**) receptor binding in P22 tumour.

The distribution of total (ET_A_+ET_B_) and ET_A_ binding tended to be higher in the tumour periphery compared to tumour centre, but this was not statistically significant (Table 2, Figure 7). The distribution of ET_B_ receptor binding was homogeneous throughout the tumour section, which represents a major difference from the discrete patches of ET_B_ binding previously observed for the HSN tumour (Bell *et al*, 1998). A high level of specific binding was observed for total (ET_A_+ET_B_) and ET_A_ receptor binding with 78 and 85% specific binding, respectively, compared with 51% specific binding for ET_B_ receptors.

## DISCUSSION

IRL 1620 caused a profound short-term reduction of blood flow in the P22 tumour, in the face of an increase in MABP, indicative of IRL 1620-induced tumour vasoconstriction. These results show that the P22 tumour vasculature is responsive to the ET_B_ receptor agonist IRL 1620 and provide indirect evidence for fully functional ET_B_ receptors on the tumour vasculature. In order to fully demonstrate that ET_B_ receptors are localised on blood vessels, double immunostaining using specific antibodies for blood vessels and ET_B_ receptors would be necessary. Profound vasoconstriction of the P22 tumour in response to IRL 1620 occurred uniformly throughout the tumour mass, at levels equivalent to or greater than that found in normal tissues. Receptor binding studies revealed existence of both ET_A_ and ET_B_ receptors in the P22 tumour and showed that ET_A_ receptors are more abundant than ET_B_ receptors, which may be significant for tumour growth. The activation of ET_B_ receptors on endothelial cells results in vasodilation via activation of the nitric oxide pathway, so that the decrease in blood flow and increase in vascular resistance in the P22 tumour following treatment with IRL 1620 indicate that the predominant vasoactive component of ET_B_ receptors is localised on contractile elements rather than on endothelial cells. IRL 1620 caused vasoconstriction in skeletal muscle, kidney and small intestine, as well as in tumour, but did not affect blood flow in other normal tissues such as skin, brain, heart and spleen. The relatively short duration of vasoconstriction in both tumour and normal tissues is presumably a reflection of both rapid plasma clearance and receptor binding kinetics in the different tissues.

In our previous studies investigating the blood flow-modifying effects of endothelin receptor ligands, such as ET-1 itself, sarafotoxin and IRL 1620 in the HSN tumour model, IRL 1620 was the most promising for selective reduction in tumour blood flow with minimal effects on systemic blood pressure and blood flow in the majority of normal tissues (Bell *et al*, 1995, 1999). In the present study, the profound vasoconstrictive capacity of IRL 1620 was demonstrated in a second tumour type and a full time course of events determined. In addition, some differences between the responses in the two model systems can be described. In contrast to responses of normal tissues of CBH/CBi rats to the action of 3 nmol kg^−1^ IRL 1620, where reduced blood flow was observed only in small intestine (Bell *et al*, 1999), the same dose of IRL 1620 in the current study using BDIX rats induced a significant blood flow reduction in kidney and skeletal muscle, as well as small intestine. This may be explained by a complex dose response for IRL 1620 in several tissues, as observed in CBH/CBi rats (Bell *et al*, 1999). For example, 1 nmol kg^−1^ IRL 1620 caused a significant reduction in blood flow rate to skeletal muscle despite no effect of 3 nmol kg^−1^. This bell-shaped dose response pattern is most likely due to desensitisation of receptors at higher doses (Bell *et al*, 1999) and the difference between skeletal muscle response in CBH/CBi rats and BDIX rats to 3 nmol kg^−1^ IRL 1620 is therefore likely to reflect differences in doses at which this desensitisation occurs, which may in turn reflect differences in receptor numbers. In addition, this strain difference in skeletal muscle response is likely to explain the markedly greater hypertensive effect of IRL 1620 in BDIX rats compared with that for CBH/CBi rats. In the current study, kidney blood flow was reduced to a similar level as that in tumours (despite a somewhat different time course), suggesting that vasoconstriction in kidney is mediated primarily through the ET_B_ receptor. This was also suggested by Just *et al* (2004), although there is also evidence for some involvement of ET_A_ receptors (Matsuura *et al* 1996; Ozawa *et al* 2003). In the present study, the response of small intestine to IRL 1620 was also vasoconstrictive, again indicating that the predominant responsive ET_B_ receptors in this tissue are on contractile elements of the blood vessels. A selective blood flow decrease in kidney and small intestine might be used therapeutically in combination with chemotherapeutic drugs such as vincristine and 5-fluorouracil, which are primarily toxic to the gastrointestinal tract or cisplatin, which is toxic to kidney. In these types of combination, IRL 1620 could be used to divert blood away from the sensitive tissues and thus lower the side effects of such treatment.

In a rat breast tumour model, employing a radioactive microsphere technique, treatment of animals with ET-1 resulted in increased tumour blood flow compared to normal breast tissue in normal rats. This increase in tumour blood flow was attenuated by pretreatment with the ET_B_ receptor antagonist BQ788 (Rai and Gulati, 2003). Therefore, the authors suggested that ET_B_ receptor agonists could be used to increase blood flow selectively to breast tumour and consequently increase delivery of anticancer drugs. This suggestion is in contrast to our current study on the P22 tumour and our previous study on the HSN tumour (Bell *et al*, 1999), where IRL 1620 decreased blood flow. Indeed, it is possible to explain the increase in tumour blood flow induced by ET-1 in the Rai study without invoking a vasodilatory role for ET_B_ receptors on the tumour vasculature. This could occur as a result of ET-1-induced hypertension causing an increase in tumour perfusion pressure combined with a relative lack of constrictive ET receptors on the tumour vasculature. In our previous study on HSN and P22 tumours, blood flow to tumours was unmodified by treatment with ET-1 (Bell *et al*, 1996). Although this difference from the Rai study is presumably due to different complements of ET receptors on the different tissues of the various rat strains, without more specific information it is not possible to say whether differences in the receptor complement on the vasculature of tumours is more important than that on the vasculature of normal tissues, the latter having a profound influence on systemic blood pressure response and, therefore, perfusion pressure to the tumour.

To assess the regional changes in tumour blood flow after treatment with IRL 1620, we used tumour uptake of ^14^C-IAP followed by autoradiography. Blood flow was homogeneously reduced throughout the tumour section. This is important information, since angiotensin II, for example, reduced tumour blood flow only in periphery, with no changes in the blood flow in the central region of the tumour (Tozer *et al*, 1996). This is probably due to the difference in receptor distribution. In the case of angiotensin II receptors, 10% more binding sites for angiotensin II were demonstrated at the periphery of P22 tumours than at the centre (Tozer *et al*, 1996) compared to homogeneous distribution of ET_B_ receptors in the current study. This suggests that ET_B_ receptors are located on the contractile elements of microvessels in the centre of the P22 tumour. Indeed, pericytes are commonly found, in close association with microvessels, in the centre of these tumours (GM Tozer, unpublished data).

Receptor binding studies on cryostat sections of the same tumours as used in the blood flow analysis showed that the P22 tumour possesses ET_A_, as well as ET_B_ receptors, with binding sites distributed relatively homogeneously across the tumour section. A high degree of specific binding, in the region of 85% of the total binding, was observed when ET_A_+ET_B_ receptors were observed together and when ET_A_ receptor binding was performed (in the presence of the ET_B_ antagonist BQ788). In contrast, a much lower intensity of ET_B_ receptors was observed in P22 tumour sections (in the presence of the ET_A_ antagonist BQ610), which corresponded to a lower level of specific binding (51%). Previous workers have reported the binding kinetics of ET-1 at ET_A_ and ET_B_ receptors, and have observed the dissociation constants (*K*_d_) of the ligand to be similar at each receptor (Ohlstein *et al*, 1996). This implies that the difference in the level of specific binding represents a higher density of ET_A_ receptors than ET_B_ receptors in the P22 tumour, rather than merely reflecting differences in the selectivity and affinity of the ligand for each receptor subtype.

ET_B_ receptor binding in the rat HSN tumour was concentrated in small regions of intense binding (Bell *et al*, 1998), which suggested specific vascular localisation. However, the ET_B_ receptor distribution in P22 tumours was homogeneous and suggests that ET_B_ receptors are localised on tumour cells as well as on the vasculature in this tumour type. Similarly, in human glioblastoma tumours, the pattern of ET_B_ receptor distribution was homogeneous suggesting localisation on cancer cells, whereas ET_A_ receptors were highly expressed in glioblastoma vessels and in some scattered glioblastoma areas (Egidy *et al*, 2000). Despite the difference in the ET_B_ receptor binding site distribution, vascular resistance in HSN and P22 tumours was increased to approximately the same degree. Over the whole tumour sections, specific ET_B_ receptor binding in the P22 tumour was 1.7 fmol mg^−1^ (Table 2) compared to 0.7 fmol mg^−1^ for the HSN tumour (Bell *et al*, 1998). Considering the homogeneous binding distribution in the P22 tumour, this figure may be a reasonable reflection of specific binding on the blood vessels. However, the heterogeneous binding in the HSN tumour suggests that specific binding on the blood vessels of this tumour may be considerably higher than the average value suggests. In view of this, the blood flow response of the two tumour types is reasonably compatible with the binding results. Considering these results, methods for measuring specific receptor densities in human tumours, either from biopsies or using imaging techniques, appear useful for predicting vascular response to vasoactive agents.

In most human tumours investigated so far, both ET receptor subtypes have been identified (Nelson *et al*, 2003), although pharmacological manipulation of the endothelin axis for cancer therapy has concentrated on the potential for ET_A_ antagonists (Nelson, 2001; Nelson *et al*, 2003; Sonveaux *et al*, 2004). Our studies suggest that the ET_B_ agonist, IRL 1620, would be useful for short-term blood flow reduction in tumours expressing the ET_B_ receptor and, due to its constrictive effect, IRL 1620 may also have antiangiogenic properties. It would be important to determine the effects of this ET-1 agonist in tumours that endogenously produce high levels of ET-1 such as prostate, breast and ovarian cancers (Nelson *et al*, 2003; Grimshaw *et al*, 2004). Although it might be expected that exogenous administration of an agonist would have little effect under these conditions, this is not necessarily the case, considering that ET receptors are often upregulated in concert with their ligands (Grimshaw *et al*, 2004).

In conclusion, our results show that fully functional ET_B_ receptors are expressed on the vasculature of at least two different tumours, leading to a relatively selective and profound short-term reduction of tumour blood flow, following administration of the ET_B_ receptor agonist, IRL 1620. Expression levels of ET_B_ receptors on the tumour vasculature could be useful for predicting which tumours are likely to respond to IRL 1620. The vasoconstriction produced in the P22 and HSN tumours was equivalent to or greater than that found for normal tissues and to our knowledge, this is a unique feature of IRL 1620 compared to other vasoconstrictors, which tend to be less effective against tumour than normal blood vessels. The activation of ET_B_ receptors with selective agonists such as IRL 1620 is therefore promising, with the proviso that normal kidney, intestine and skeletal muscle are also affected. Distribution of ET_B_ binding in the P22 tumour suggested ET_B_ receptor localisation on tumour cells as well as on the vasculature, which may also be significant for tumour growth. Blood flow results suggested that the vascular component of ET_B_ receptors is localised on contractile elements rather than on endothelial cells. Considering the profound vasoconstrictive effect of a single dose of IRL 1620 in tumours, further studies are warranted to determine its effects in a repeated dosing schedule on tumour angiogenesis and growth, where selectivity of vascular effect for tumours may not be critical. In addition, further studies using specific ET-1 receptor agonists and antagonists should provide more insight into the role of the endothelin axis in the tumour vasculature, leading to the development of specific vascular-targeted therapies.

## Figures and Tables

**Table 1:** Tumour and tissue blood flow in saline-treated BDIX rats bearing P22 tumour

**Tissue**	**Blood flow (AM±s.e.^a^ (ml g^−1^** **min^−1^))**	***P***-**value compared to tumour blood flow**
Tumour	0.42±0.02	
Skin overlying tumour	0.20±0.02	<0.05
Contralateral skin	0.14±0.01	<0.05
Skeletal muscle	0.41±0.05	>0.05
Brain	1.43±0.10	<0.05
Heart	3.44±0.38	<0.05
Kidney	4.72±0.51	<0.05
Spleen	2.42±0.24	<0.05
Small intestine	1.22±0.09	<0.05

a Arithmetic mean±standard error of the mean.

**Table 2:** Receptor binding in P22 tumour sections

		**Specific receptor binding**					
		**Whole^a^**		**Periphery^b^**		**Centre^c^**	
**Binding site**	***n***	**AM±s.e.^d^ (fmol mg^−1^ protein)**	**% of total binding**	**AM±s.e. (fmol mg^−1^ protein)**	**% of total binding**	**AM±s.e. (fmol mg^−1^ protein)**	**% of total binding**
ET_A_+ET_B_^e^	12	6.6±1.8	78	7.1±2.1	78	6.3±0.9	79
ET_A_^f^	12	6.7±2.0	85	7.3±2.1	85	6.1±2.0	85
ET_B_^g^	12	1.7±0.4	51	1.7±0.4	49	1.6±0.4	51

ET_A_=endothelin A; ET_B_=endothelin B.

a Binding of ligands to whole tumour section.

b Binding of ligands to peripheral tumour region.

c Binding of ligands to central tumour region.

d Arithmetic mean±standard error of the mean.

e Binding of ^125^I-ET-1 to tumour section.

f Binding of ^125^I-ET-1 with the addition of BQ-788 to tumour section.

g Binding of ^125^I-ET-1 with the addition of BQ-610 to tumour section.
